# Optic phonons and anisotropic thermal conductivity in hexagonal Ge_2_Sb_2_Te_5_

**DOI:** 10.1038/srep37076

**Published:** 2016-11-16

**Authors:** Saikat Mukhopadhyay, Lucas Lindsay, David J. Singh

**Affiliations:** 1 Materials Science and Technology Division, Oak Ridge National Laboratory, Oak Ridge, TN 37831 USA; 2 Department of Physics and Astronomy, University of Missouri, Columbia, MO 65211-7010 USA

## Abstract

The lattice thermal conductivity (κ) of hexagonal Ge_2_Sb_2_Te_5_ (h-GST) is studied via direct first-principles calculations. We find significant intrinsic anisotropy (*κ*_*a*_/*κ*_*c*_~2) of κ in bulk h-GST, with the dominant contribution to κ from optic phonons, ~75%. This is extremely unusual as the acoustic phonon modes are the majority heat carriers in typical semiconductors and insulators. The anisotropy derives from varying bonding along different crystal directions, specifically from weak interlayer bonding along the *c-*axis, which gives anisotropic phonon dispersions. The phonon spectrum of h-GST has very dispersive optic branches with higher group velocities along the *a-*axis as compared to flat optic bands along the *c-*axis. The large optic mode contributions to the thermal conductivity in low-κ h-GST is unusual, and development of fundamental physical understanding of these contributions may be critical to better understanding of thermal conduction in other complex layered materials.

Phase change memory devices exploit thermally induced transformations between different phases of phase change materials (PCMs) where thermal and thermoelectric properties of PCMs are of central importance. Ge_2_Sb_2_Te_5_ (GST), a prototypical phase change material, has been widely studied^1^,^2^,^3^,^4^,^5^,^6^,^7^,^8^,^9^,^10^ in this context due to its fast switching speed^11^ and large changes in electrical and optical properties upon phase transformation. Recently, Lee *et al*.^5^ reported anisotropic thermal conduction of GST thin films with *in-plane* film values significantly different from *cross-plane* film values for cubic and hexagonal phases and noted a varying conductivity with annealing time of the samples. They reported that cross-film conduction is favored by up to 54% due to thermal resistance of amorphous regions near grain boundaries^5^. All of the GST phases (cubic, hexagonal and amorphous) exhibit low thermal conductivity, and it has been shown that the thermal properties in thin film samples vary significantly with sample thickness attributed to boundary scattering and microstructure^8^. As thin film samples have varying degrees of extrinsic defects and microstructure, it is not clear whether there is *intrinsic* thermal conductivity anisotropy in bulk h-GST, anisotropy due solely to the hexagonal structure. In addition, h-GST has been shown to exhibit good thermoelectric properties^12^,^13^,^14^, though thermal conductivity studies particularly in bulk h-GST are limited.

Identifying mechanisms for obtaining low thermal conductivity and modification of materials to obtain it, *e.g.*, by nanostructuring and alloying, is an important part of thermoelectrics research. This typically focusses on scattering of heat carrying acoustic phonons^15^. Optic phonon contributions are often negligible due to their typically low group velocities and short lifetimes^16^,^17^, even in cases where they comprise a large fraction of the total modes in complex crystals. An exception is the nuclear fuel, UO_2_, where the longitudinal optic branch was reported to be important^18^. Recently it has been shown that optic mode contributions can become important in other materials of interest for thermoelectric applications, *e.g.*, PbTe (22%) and PbSe (25%)^19^, Mg_2_Si (30%) and Mg_2_Sn (18%)^20^. Here we examine the lattice dynamical properties of h-GST from first principles and connect these to its lattice thermal conductivity by direct calculations. We find κ values consistent with measured data. Importantly we find that in h-GST the optic phonons are the *dominant* heat carriers (~75%) at room temperature, and that these modes significantly affect the behavior of κ for different transport directions. We find significant intrinsic thermal conductivity anisotropy arising from the hexagonal structure and optical phonon contributions. This anisotropy may be of critical importance in phase change devices that contain h-GST in the crystalline phase with preferential orientations for crystallization.

## Results

**Figure 1:**
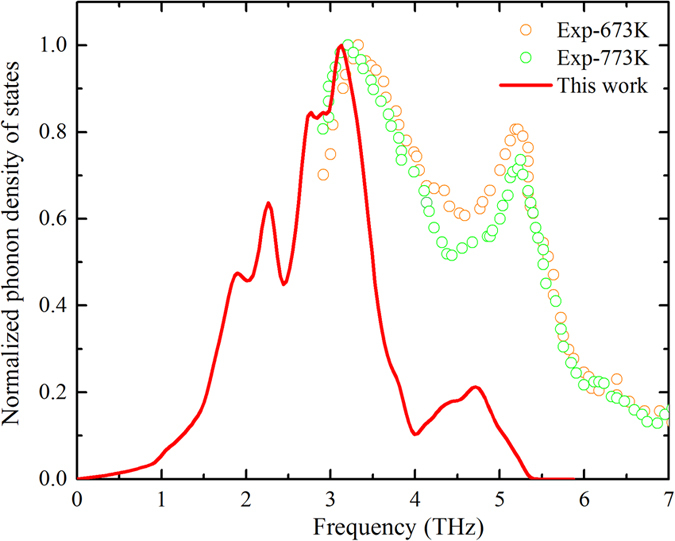
Comparison of phonon density of states (normalized) at Γ along with the measured Raman spectra 21 at 673 K (orange circles) and 773 K (green circles).

We compare the calculated phonon density of states (PHDOS) with the measured Raman spectra^21^ of polycrystalline hexagonal GST films at 673 K and 773 K in Fig. 1. Two distinct peaks were measured at 3.2 THz (peak-A) and 5.1 THz (peak-B) and also appear in our calculation. Peak-A, reported to be the vibration of the heteropolar bond in the tetrahedral GeTe^4^ and pyramidal SbTe^3^ 21, compares well (3.11 THz) with our calculation, however, the frequency of peak-B is underestimated (4.79 THz) by our calculation. A similar underestimation of the peak-B frequency (5.01 THz) was also reported in a previous theoretical work by Sosso *et al*.^22^ Regardless, this demonstrates the relative accuracy of the harmonic force constants in our calculations. We also note that the calculated PHDOS here compares well with the previously reported PHDOS from a first-principles calculation^23^ with slightly different acoustic velocities. These small variations likely arise from different DFT approximations employed [LDA^23^ vs. GGA (this work)] as LDA (GGA) typically gives shorter (longer) bonds, thus higher (lower) sound velocities. However, our calculated sound speed of the longitudinal acoustic mode 3.7 km/s (3.2 km/s) along *a*-axis (*c-*axis) compares well that measured in experiment (3.3 km/s)^6^.

**Figure 2:**
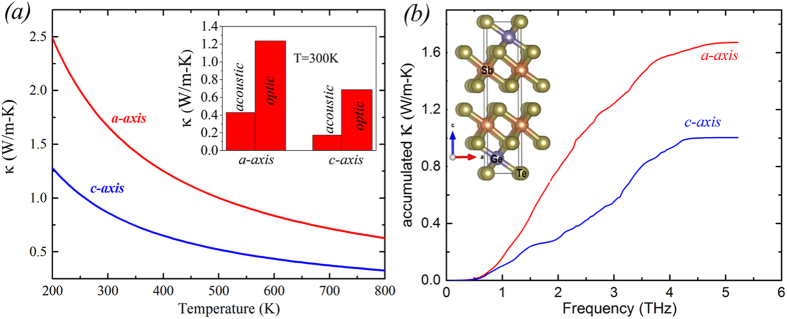
(**a**) Lattice thermal conductivity and (**b**) accumulated thermal conductivity with phonon frequency of h-GST along a-axis (red) and c-axis (blue). The contributions from optic and acoustic modes to total κ at 300 K along a- and c-axis are shown in the inset of Fig. 2(a). A schematic of the unit cell of h-GST is also shown in the inset of Fig. 2(b).

The calculated κ as a function of temperature is given in Fig. 2(a), which clearly shows a strongly anisotropic κ along the two crystal directions: *a*- and *c*-axis. This is of potential importance for devices with h-GST (the crystalline phase in devices can be either h-GST or cubic GST) because strain, thermal gradients, directional growth, and interaction with substrates and interfaces can all lead to preferential alignment of the crystal axis, which in combination with the thermal conductivity anisotropy will affect device behavior. Furthermore, if controlled, it may provide another pathway for improving performance. As shown in Fig. 2(a), κ at 300 K for h-GST along the *a-*axis (1.67 W/m-K) is nearly twice of that along the *c*-axis (0.86 W/m-K). This anisotropy (*κ*_*a*_/*κ*_*c*_~2) persists throughout the entire temperature-range of this study. The magnitude of the anisotropy is similar to that found for layered Bi_2_Te_3_^24^,^25^,^26^ and higher than that of manganese silicide (HMS)^27^. While significant bonding anisotropy was held responsible for the anisotropic κ in Bi_2_Te_3_^28^ with van der Waals gaps in the low κ direction, a low-energy (<10 meV) twisting phonon and anisotropic group velocity of the optic phonons were found responsible for the observed κ anisotropy in HMS^27^. We note that Lee *et al*.^5^ measured an anisotropic κ favoring cross-film conductivity in polycrystalline h-GST thin films. Crystal orientation relative to the film may not be commensurate with *a-* and *c-*axis alignment and the anisotropy in experiment is attributed to amorphous regions near columnar grain structures in the films. The anisotropy reported here is due to intrinsic properties of the hexagonal crystal structure, thus direct comparison of these anisotropy mechanisms is not possible. In the following, we elaborate on the origin of the intrinsic anisotropy of κ in h-GST, and on the surprisingly large κ contribution from the optic phonon modes.

Firstly, the calculated κ for both crystal directions in Fig. 2(a) give the typical *T*^−1^ behavior, characteristic of Umklapp scattering, up to the highest temperature considered (*T* = 800 K). This is at variance with roughly *T* independent thermal conductivity measured by Lyeo *et al*.^6^. Further, the reported thermal conductivity (1.53 W/m-K)^6^ of thin film (270 nm) h-GST at 673 K is significantly higher than the calculated κ values given here: 0.75 and 0.39 W/m-K for the *a*- and *c*-axis, respectively. This discrepancy is likely due to a significant electronic contribution to the total thermal conductivity as h-GST has a relatively small band gap [~0.5 eV]^29^ in combination with a low lattice thermal conductivity. Using the Weidemann-Franz law coupled with electrical conductivity measurements, Lyeo, *et al*.^6^, estimated the electronic contribution to the total thermal conductivity was ~70% giving an estimated lattice contribution of 0.43 W/m-K at 673 K. Increasing electronic contribution to the total thermal conductivity would compensate the decreasing lattice thermal conductivity resulting from enhanced phonon-phonon scattering with increasing temperature, resulting in the *T* independent thermal conductivity behavior observed for h-GST in experiment. The calculated average κ (0.63 W/m-K) overestimates the measured value likely due to extrinsic scattering, *e.g.*, grain boundaries and impurities. Further, the thermal conductivity of thin film h-GST is sensitive to sample thickness^8^. For instance, Reifenberg *et al*. measured a thermal conductivity (electron + phonon) of 350 nm h-GST thin films that was more than twice of that in samples with thickness of 60 nm^8^. Given the thickness of the samples in ref. 6 were 270 nm, this may also explain the overestimated calculated κ as compared to measurement. We estimated the effect of grain boundaries on κ using a simple empirical model for diffuse boundary scattering and found an increase of 11% in κ for a boundary scattering length of 350 nm as compared to that for a scattering length of 60 nm. We note that our calculations do not include the effects of boundary scattering on the electronic contribution to the thermal conductivity. Nevertheless, the focus of this study is to understand features of the intrinsic lattice thermal conductivity of h-GST, therefore, extrinsic scattering and the electronic contribution to κ are not considered further.

**Figure 3:**
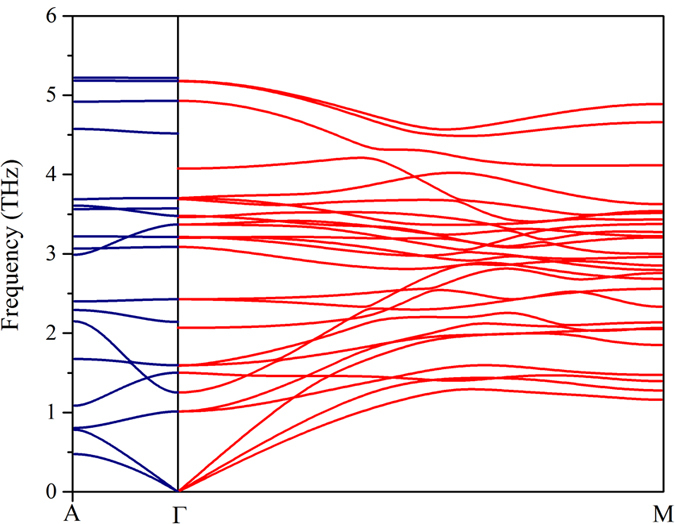
Phonon dispersions of h-GST along the *a-*axis (red) and the *c-*axis (blue).

The accumulated thermal conductivity with phonon frequency [Fig. 2(b)] for the *a-* and *c-*axis demonstrates isotropic κ up to ~0.8 THz with a significant anisotropy in accumulated κ above this. This is precisely where the acoustic and optic phonon branches start to cross and the acoustic branches reach the zone boundary along the *c*-axis direction. Figure 3 compares the phonon dispersions for the *a*-axis (Γ → M direction) and *c*-axis (Γ → A direction) for h-GST. Firstly, we note that the overall acoustic frequency scale is comparable to other thermoelectric materials (lower than PbTe, Mg_2_Si and Mg_2_Sn; similar to Bi_2_Te_3_), partly due to the large masses of the constituent atoms and partly because of the similarity in bonding to PbTe and Bi_2_Te_3_. This gives lower velocity acoustic phonons and enhanced phonon-phonon scattering. The slopes of the dispersion curves give the phonon velocities, important in determining κ. More importantly, we note that the total κ consists of a dominant contribution from the optic modes for both crystal directions. While the conventional heat carriers, acoustic modes, give 25% (20%) of total κ along the *a*-axis (*c*-axis), 75% (80%) of the total κ is carried by optic modes [inset in Fig. 2(a)]. This is unconventional as optic mode contributions are not reported to give more than 25%^19^,^20^ of the total κ, and are typically much smaller. This warrants further investigations of thermal transport in low-κ materials with many low frequency optic branches.

The phonon dispersion of h-GST shows an inherent anisotropy with highly dispersive optic branches (large group velocities) along the *a*-axis in contrast to flat optic branches along the *c*-axis. Further, the acoustic braches along the *c-*axis do not cross the low-lying optic modes apart from the zone boundary, while the acoustic branches along the *a*-axis undergo multiple crossings. This is largely a consequence of the smaller Brillouin zone along the *c*-axis. This is similar to GaAs/AlAs superlattices where the constituting *n*-units of bulk GaAs and AlAs were arranged along the growth axis so that the edge of the Brillouin zone along the growth direction is 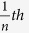 as far from the zone center as it is along *a-* and *b-*axis^30^. This was reported to significantly reduce the group velocities of optic modes along the *c*-axis. Importantly, the acoustic mode velocities near the zone center have little anisotropy and therefore elastic differences are not the origin of the thermal conductivity anisotropy, as will be discussed in more detail below.

**Figure 4:**
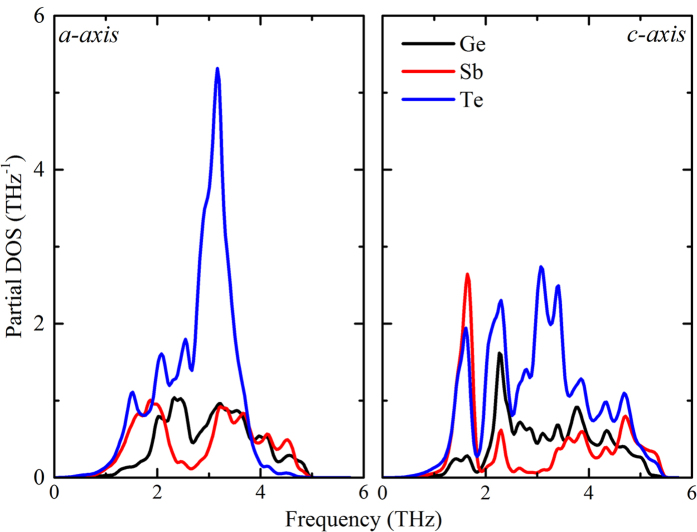
Phonon density of states projected on individual atoms of h-GST along a-axis and c-axis.

To further evaluate the character of the phonon dispersions the projected phonon densities of states for the various atomic species are given in Fig. 4. The lowest frequency modes are predominantly from vibrations of the heavy Sb and Te atoms, with strong contributions from Ge-Te modes starting at ~2 THz. This leads to two prominent peaks in the density of states, the first of Sb-Te character from ~1–1.5 THz, and the second of Ge-Te character from ~ 2–2.5 THz. The higher optic branches have mixed Ge-Sb-Te character extending all the way to the top of the spectrum. It is striking to note the differences in projected phonon DOS along *a-* and *c-*directions. Although in the low frequency region, the vibrational modes (acoustic modes) along these crystal directions are similar, the vibrational modes in the higher frequency region (optic modes) are very different. While the optic modes along the *c-*axis are mostly stretching and anti-stretching modes consisting of Ge-Te and Sb-Te atoms, the optic modes along *a-*axis are notably more complex and include torsional and twisting modes in addition to the stretching and anti-stretching modes. This is due to the significantly stronger contribution from Te and Sb atoms along the *a-*axis at frequencies higher than 2.5 THz (Fig. 4).

While acoustic velocities are generally considered to be central to conductivity anisotropy, especially for low-κ systems like h-GST, we find no significant anisotropy in the group velocities. We calculated the average acoustic sound velocity along each direction using: 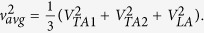 Along the *a*-axis *v*_*avg*_ = 2.87 km/s compared to 2.75 km/s along the *c*-axis, failing to explain the noted anisotropy of κ in Fig. 2. Therefore, it can be anticipated that the higher group velocities of the more dispersive optic bands along the *a*-axis as compared to the flat bands along the *c*-axis are responsible for the κ anisotropy in h-GST, especially since these modes govern the thermal transport behavior in this system.

**Figure 5:**
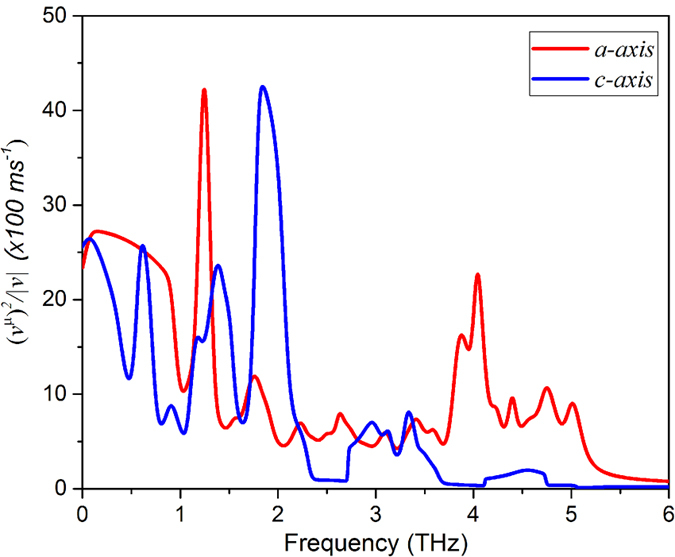
Calculated average group velocity components along the a- and c- axes as a function of frequency.

In order to support the above statement, we plot the frequency-dependent averages of each component of the group velocity in Fig. 5. This is similar to the density regression estimates over all branches along the *a-* and *c-*axis^27^:


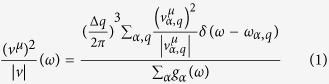


where *α* sums over all modes, *q* sums over all lattice vectors in the Brillouin zone, 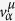 is the group velocity along the μ-direction and *g_α_*(*ω*) is phonon density of states.

As can be found in Fig. 5, the average group velocities of the optical modes (>1 THz) are appreciable in both crystal directions, and surprisingly the group velocity of the low lying optic branches surpasses that of the acoustic branches. We note that even the highest frequency optic phonons in the *a*-direction are significantly dispersive. Often optic modes are not considered as heat carriers because their velocities are small compared to their acoustic counterparts. In fact, we find the opposite is true in h-GST: the optic modes contribute 75% (80%) to κ along the *a*-axis (*c*-axis). Not only that, we also find notable anisotropy in group velocities of optic modes along the two crystal directions. While the group velocities of low lying TO modes along the *a*-axis dominate over that along the *c*-axis in the frequency range 1–1.5 THz, the opposite occurs around 2 THz. Moreover, the group velocities of the highest-frequency optic modes (>3.5 THz) along the *c*-axis are very small compared to that along the *a*-axis. Given that the anisotropy in the acoustic group velocity is not significant, these may very well explain the noted κ anisotropy provided the anharmonicities (as measured by mode Grüneisen parameters here) associated with these optic modes are not so high as to completely suppress the phonon lifetimes, and thus their contributions to κ.

**Figure 6:**
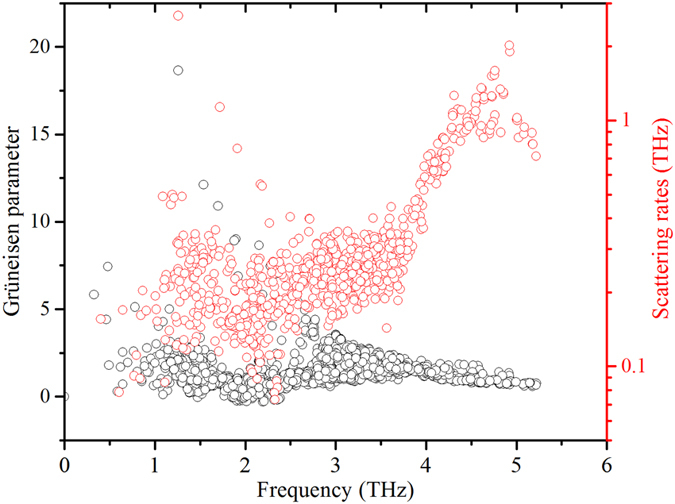
Frequency dependent mode Grüneisen parameters and scattering rates derived from third-order anharmonic force constants.

We show the frequency dependent mode Grüneisen parameters derived from third-order anharmonic force constants^28^ as implemented in Phono3py^31^ in Fig. 6. We note that mode Grüneisen parameters are independent of crystal direction as well as the scattering rates determined here within the relaxation time approximation. Although the mode Grüneisen parameters vary over a wide range (~−1.0 to 18.0), the average over the whole frequency range is calculated to be 1.46. This is less than the average mode Grüneisen parameter of the acoustic modes of PbTe, PbSe and PbS^32^ despite the fact that the total thermal conductivity at 300 K is comparable. We note that the Grüneisen parameters of the high frequency modes are similar to or less than the lower frequency acoustic modes. Therefore, as far as anharmonicity is concerned, contributions to κ from optic modes are expected to be similar to or higher than those of acoustic modes with comparable velocities.

**Figure 7:**
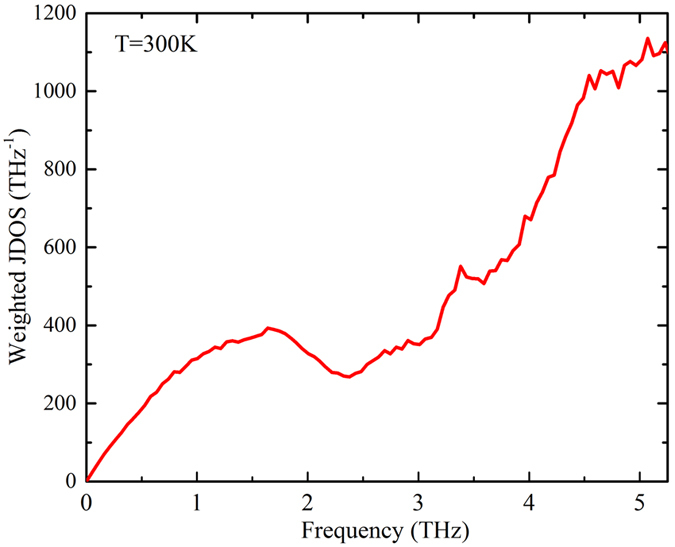
Frequency dependent weighted joint density of states.

The Grüneisen parameters, however, fail to explain the increase in scattering rates (Fig. 6) and the saturation in the accumulated κ along the *a*-axis above 3.8 THz (Fig. 2b) despite these branches being significantly dispersive. Given the average mode Grüneisen parameters remain nearly constant in this frequency range and they are independent of crystal direction, one would expect similar scattering rates and significant contributions from the dispersive high frequency branches and further κ anisotropy. This can be explained in terms of the phase space available for three phonon scattering processes in this frequency range as shown in the joint density of states^31^ in Fig. 7 (see SI for details). The joint density of states is weighted by the Bose-distribution function of the phonon modes involved in the scattering processes at 300 K. The phase space for three-phonon scattering processes increases dramatically for frequencies higher than 3.8 THz, thus the optic phonons in this frequency range have significantly higher scattering rates [Fig. 6], which suppresses their contribution to κ even though the group velocities (and anisotropy) of these modes are significant. Therefore, we conclude that the thermal conductivity anisotropy in h-GST is mainly due to the dispersive optic branches with frequency less than 3.8 THz along the *a*-axis compared to the flat optic branches with lower group velocities along the *c*-axis. The differences in optic mode averaged velocities in a material with similar acoustic velocities can be understood in terms of zone folding effects. In particular, the zone folding along the long *c*-axis leads to more intersections with zone boundaries in this direction. Symmetry requires that non-degenerate modes have zero slopes when touching the boundary, *i.e.*, zero *c*-direction velocities, but does not impose constraints on the velocities transverse to the boundary. This zone folding effect leads to a material that exhibits thermal anisotropy even though it is mechanically rather isotropic (*i.e.*, the zone center sound velocities are nearly isotropic).

**Figure 8:**
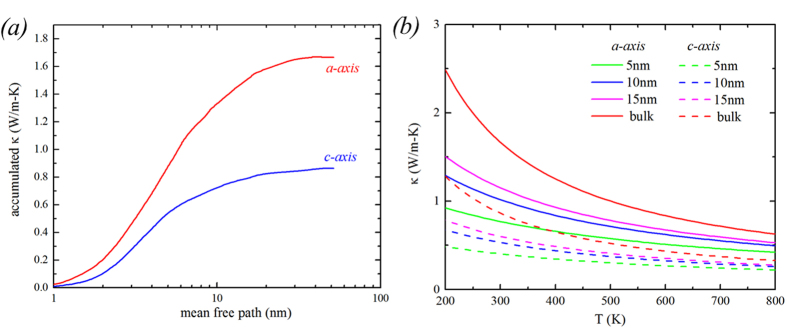
(**a**) Accumulated κ as a function of phonon mean free path. (**b**) κ as a function of temperature for various grain sizes. The green, blue and magenta curves represent κ for grain sizes of 5 nm, 10 nm and 15 nm, respectively. The dashed and solid lines are for κ along c-axis and a-axis, respectively. The red lines represent bulk κ.

Finally, accumulated κ as a function of phonon mean free path (λ_ph_) provides insight into heat conduction in nanostructured materials. Figure 8(a) clearly shows that most of the heat is carried by vibrational modes with λ_ph_ ranging from 1–30 *nm* in h-GST crystals at 300 K. On the basis of this knowledge, we calculated κ at various temperatures using various grain sizes to examine the nanostructuring effect on κ in this system (Fig. 8b). We find that a reduction of κ as much as 54% can be achieved for grain size of 5 *nm*. Even for a grain size of 15 *nm* 31% reduction of κ compared to its bulk counterpart can be achieved. The effect of nanostructuring becomes more prominent at lower temperature where intrinsic λ_ph_ values become larger. For example, at 200 K, for a grain size of 15 *nm*, κ drops 65% along *a-axis* and 63% along *c-axis*.

## Discussion

We presented phonon dispersions and thermal conductivity calculations for h-GST. Although h-GST is a relatively isotropic material from a sound velocity perspective, it has a significantly anisotropic thermal conductivity. The reason for this is zone folding due to *c*-axis layering. This can be regarded as similar to a superlattice phonon scattering mechanism and is reflected in the fact that the anisotropy in the accumulation function starts only in the energy range of the optic branches. This effect is important because the low lying high velocity optic modes contribute significantly to thermal conductivity in h-GST, particularly for the in-plane direction. Intrinsic anisotropy in the thermal conductivity is potentially of interest for devices in cases where the crystalline h-GST phase is used, and the importance of optic phonon contributions to thermal transport may have significant impact on thermoelectric efficiency in materials with complicated phonon dispersions.

## Methods

Calculations were done with the projector augmented wave (PAW)^33^,^34^ method using experimental lattice parameters^35^ and the Generalized Gradient Approximation (GGA) of Perdew, Burke and Ernzerhof^36^,^37^ as implemented in VASP^38^,^39^. The internal atomic coordinates were determined by total energy minimization. A 12 × 12 × 4 Monkhorst-Pack grid was used for sampling of the Brillouin zone with an energy cutoff of 550 eV. The convergence threshold for the energy was set to 10^−5^ eV and 10^−3^ eV/Å for its gradient. We employed a finite displacement method using the VASP-phonopy^40^ interface to calculate the harmonic force constants with higher convergence thresholds; 10^−8^ eV and 10^−8^ eV/Å for the energy and its gradient, respectively. Born effective charges were calculated using density functional perturbation theory. The third order anharmonic force constants were calculated using VASP-phono3py^31^ with a 2 × 2 × 2 supercell incorporating interactions out to 5^th^ nearest neighbors. Finally, we calculated thermal conductivity by explicitly solving the phonon Boltzmann transport equation using 12 × 12 × 4 integration meshes. A tetrahedron method was employed for the calculation of the imaginary parts of self-energies. We note that the DFT and κ calculations changed little when increasing integration meshes to 12 × 12 × 8 and 12 × 12 × 12. Details of the method can be found elsewhere^31^.

Two different crystal structures have been reported for h-GST. We considered the h-GST structures given by Kooi *et al*.^35^ and Matsunaga *et al*.^41^,^42^, which differ in Ge-Te stacking. Kooi’s structure gives notably lower total energy (0.15 eV) and is therefore likely the correct structure, thus we use this for the κ calculations. We note that while both structures resulted in stable phonon dispersions (no imaginary modes), the lowest order TO(Γ) mode was found to be situated at higher frequency in Matsunaga’s structure [See Fig. S1 in the Supplementary Information] compared to Kooi’s structure implying sensitivity of the low frequency TO mode to the positions of Ge and Sb.

## Additional Information

**How to cite this article**: Mukhopadhyay, S. *et al*. Optic phonons and anisotropic thermal conductivity in hexagonal Ge_2_Sb_2_Te_5_. *Sci. Rep.* **6**, 37076; doi: 10.1038/srep37076 (2016).

**Publisher's note**: Springer Nature remains neutral with regard to jurisdictional claims in published maps and institutional affiliations.

## Supplementary Material

Supplementary Information
