# Supplementary material for: Optic phonons and anisotropic thermal conductivity in hexagonal Ge2Sb2Te5
Source: Sci Rep. 2016 Nov 16;6:37076. doi: 10.1038/srep37076 (PMC5111060; doi:10.1038/srep37076)
Supplement: Supplementary Information [file srep37076-s1.pdf]

Supplementary Information

For

Optic phonons and anisotropic thermal conductivity in hexagonal $Ge_2Sb_2Te_5$

Saikat Mukhopadhyay[1*], Lucas Lindsay[1], David J. Singh[2]

[1]Materials Science and Technology Division, Oak Ridge National Laboratory, Oak Ridge, TN 37831 USA

[2] Department of Physics and Astronomy, University of Missouri, Columbia, MO 65211-7010 USA

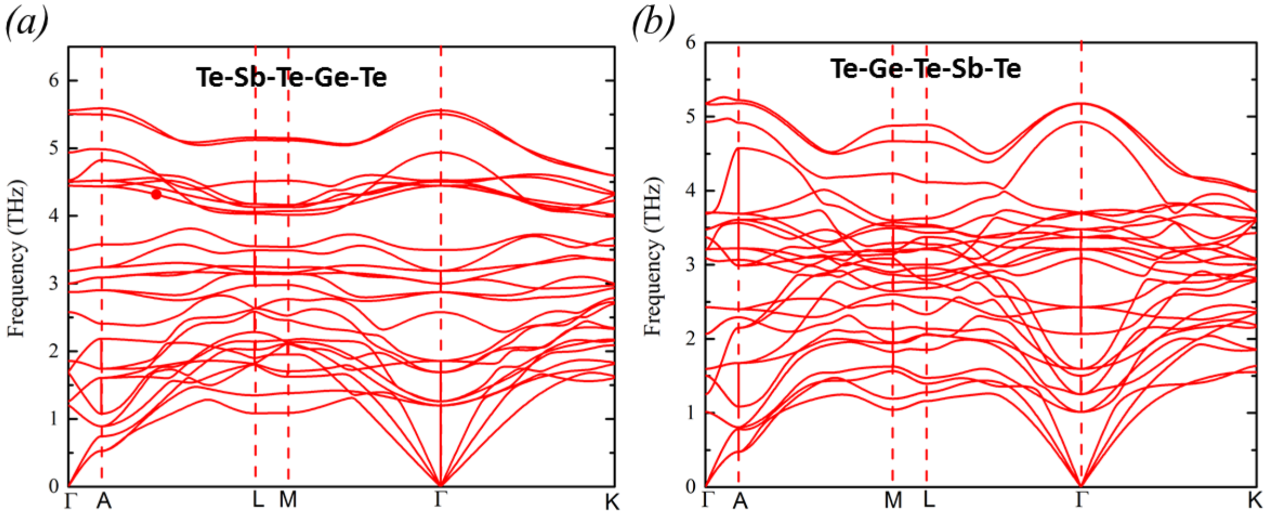

Figure S1: Phonon dispersion of h-GST for (a) Te-Sb-Te-Ge-Te and (b) Te-Ge-Te-Sb-Te stacking as given by Mantsunga[1,2] and Kooi[3], respectively.

Joint density of state (JDOS): JDOS was calculated based on the harmonic force constants using the following expressions:

$$N_2^{(1)} = \frac{1}{N}\sum_{\lambda'\lambda''} \Delta(-q+q'+q'')(n_{\lambda'} - n_{\lambda''})[\delta(\omega + \omega_{\lambda'} - \omega_{\lambda''}) - \delta(\omega - \omega_{\lambda'} + \omega_{\lambda''})]$$

$$N_2^{(2)} = \frac{1}{N}\sum_{\lambda'\lambda''} \Delta(-q+q'+q'')(n_{\lambda'} + n_{\lambda''} + 1)\delta(\omega - \omega_{\lambda'} - \omega_{\lambda''})$$

With $N_2^{(1)}$ and $N_2^{(2)}$ representing the number of scattering processes of $\delta(\omega - \omega_{\lambda_1} - \omega_{\lambda_2})$ or $\delta(\omega + \omega_{\lambda_1} - \omega_{\lambda_2})$ kind, respectively. Here, we are considering scattering of phonon λ with λ'

and λ'' with wave vectors $q, q'\ and\ q''$ and frequencies$\omega, \omega_{\lambda_1}\ and\ \omega_{\lambda_2}$, respectively. $n_{\lambda'}\ and\ n_{\lambda''}$ are the Bose distribution function for phonons $q'\ and\ q''$ calculated at T=300K.

References

[1] T. Matsunaga, R. Kojima, N. Yamada, K. Kifune, Y. Kubota, and M. Takata, Appl Phys Lett **90**, 161919 (2007).

[2] T. Matsunaga and N. Yamada, Phys Rev B **69**, 104111 (2004).

[3] B. J. Kooi and J. T. M. De Hosson, J Appl Phys **92**, 3584 (2002).
